# Functional Anatomy of Split Compound Eyes of the Whirligig Beetles *Dineutus mellyi* (Coleoptera: Gyrinidae)

**DOI:** 10.3390/insects15020122

**Published:** 2024-02-07

**Authors:** Jacob Muinde, Tian-Hao Zhang, Zu-Long Liang, Si-Pei Liu, Esther Kioko, Zheng-Zhong Huang, Si-Qin Ge

**Affiliations:** 1 Key Laboratory of Zoological Systematics and Evolution, Institute of Zoology, Chinese Academy of Sciences, Beijing 100101, China; mulwa.muinde@ioz.ac.cn (J.M.); shanezth@126.com (T.-H.Z.); liangzulong@ioz.ac.cn (Z.-L.L.); spliu@ioz.ac.cn (S.-P.L.); 2 National Museums of Kenya, Museum Hill, Nairobi P.O. Box 40658-00100, Kenya; ekioko@museums.or.ke; 3 University of Chinese Academy of Sciences, Beijing 100049, China

**Keywords:** Gyrinidae, dorsal, ventral, apposition, superposition, three-dimensional reconstruction

## Abstract

**Simple Summary:**

Whirligig beetles inhabit both fresh and brackish waters and possess a pair of split compound eyes. The dorsal eyes are situated above the water surface, whereas the ventral eyes are submerged beneath the water. Due to the difference in optical environments between water and air, we expected a difference in visual features and signal processing between the dorsal and ventral eyes. Using scanning electron microscopy, transmission electron microscopy, and microcomputed tomography, we investigated the functional modifications of different features of the split compound eyes of *Dineutus mellyi* (Coleoptera: Gyrinidae). Both dorsal and ventral parts of the split compound eyes of *D. mellyi* are the superposition type, with the ommatidium of dorsal and ventral eyes comprised of a laminated corneal lens, bullet-shaped crystalline cone, upper distal rhabdom, a clear zone and lower distal rhabdom, a basal rhabdom, and an eight retinular cell just above the basement membrane. In contrast to the dorsal eyes, ventral eyes are characterized by a higher field of vision but exhibit similar spatial resolution.

**Abstract:**

The functional anatomy of the split compound eyes of whirligig beetles *Dineutus mellyi* (Coleoptera: Gyrinidae) was examined by advanced microscopy and microcomputed tomography. We report the first 3D visualization and analysis of the split compound eyes. On average, the dorsal and ventral eyes contain 1913 ± 44.5 facets and 3099 ± 86.2 facets, respectively. The larger area of ventral eyes ensures a higher field of vision underwater. The ommatidium of the split compound eyes is made up of laminated cornea lenses that offer protection against mechanical injuries, bullet-shaped crystalline cones that guide light to the photoreceptive regions, and screening pigments that ensure directional light passage. The photoreceptive elements, made up of eight retinular cells, exhibit a tri-tiered rhabdom structure, including the upper distal rhabdom, a clear zone that ensures maximum light passage, and an enlarged lower distal rhabdom that ensures optimal photon capture.

## 1. Introduction

Whirligig beetles (Coleoptera: Gyrinidae) are among the aquatic beetles that spend most of their time gyrating on the water surface [1,2]. This behavior is attributed to hunting and feeding behaviors, whereby they prey on insects trapped in water [3]. These beetles inhabit both fresh and brackish waters and exist in either monospecific or plurispecific masses, a behavior ascribed to maximum scavenging efficiency and predator avoidance [4,5,6,7]. When they detect any threat, whirligig beetles swirl on the water surface at a very fast speed [8,9].

Perhaps the most astounding feature of the whirligig beetles is the complex eye conformation with split compound eye structures forming dorsal and ventral eyes [10,11]. Analogous to the split eyes of the four-eyed fish *Anableps anableps* that live at the water surface and the split eyes of midwater hyperiid amphipods, this phenomenon is an adaptational feature to suit the beetle’s lifestyle: the dorsal eyes of the whirligig beetles are positioned above the water surface while the ventral eyes are submerged in the water [12,13,14].

Compound eyes in insects are made up of photoreceptive units known as ommatidia, and each of the ommatidia is composed of dioptric apparatus and photoreceptive elements. The dioptric apparatus, cornea lens, and crystalline cone receive and direct light towards the photoreceptive regions that are made up of retinular cells, which absorb light signals and convert them to photoelectric signals before transmission to the central nervous system via the axons [15].

Light behaves differently in water and air, creating two different optical environments [16]. When light transitions from air to water, it undergoes refraction and water also scatters light, leading to poor visibility [17]. Therefore, due to varying light parameters in air and water, the dorsal and ventral eyes are expected to exhibit different adaptations, including in their general anatomy [18,19] and nanostructures. Salamanca & Brown (2018) reported that the dorsal eyes of whirligig beetles *Gyretes sericeus* exhibit morphological similarities to the apposition compound eyes of the diurnal insects, while the ventral eyes showed similar morphological features to the superposition eyes of the nocturnal insects [20]. Atomic force microscope images revealed distinct nanostructures on the facets of the dorsal and ventral eyes in both genera *Gyrinus* and *Orectochilus*. Specifically, the ventral facets exhibited a smooth surface, whereas the dorsal facets displayed maze-like nanostructures, which indicate the presence of anti-reflective properties, with a spectral preference within the 450–600 nm range [1]. The distinct optic neuronal transmission between the dorsal and ventral eyes suggests functional differentiation. In the adult gyrinidae beetle *Dineutus sublineatus*, the presence of the lobula plate, which is associated with hunting activities, is only observed in conjunction with the lower lobula [14]. However, the calyces, which serve as the primary input region of the mushroom body, are more robust in *D. sublineatus* compared to other aquatic insects. Furthermore, they are exclusively innervated by visual neurons from the medulla of the dorsal eye optic lobes [21]. These findings indicate that the ventral eyes are primarily involved in prey capture, while the dorsal eyes are associated with visual place memory [14,21].

In this study, we present a detailed description and analysis of the basic organization of the split eyes of the whirligig beetle *Dineutus mellyi* by identifying the functionally relevant features in the ommatidial structure through scanning electron microscopy and transmission electron microscopy. In addition, we visualize the internal morphology of the split compound eyes through microcomputed tomography aiming at determining the relative positions of the different features that make up the split compound eyes. This study provides new details about the functional anatomy of split compound eyes with implications for visual ecology.

## 2. Materials and Methods

### 2.1. Specimen Collection

Adult male and female *D. mellyi* were collected from Mt. Danxia (25°1′28″ N, 113°39′9″ E, 120 m) in Guangdong Province in December 2020. The samples were kept in plastic cases filled halfway with water and moved to the laboratory at the Institute of Zoology, Beijing. In the laboratory, the samples were maintained in an aquarium tank (100 × 30 × 40 cm) with a controlled water temperature of 20–25 °C and a photoperiod of 15:9 h. The beetles were fed daily using frozen *Drosophila melanogaster* and fresh bloodworms (*Chironomus* larvae).

### 2.2. Scanning Electron Microscopy

For examination of the external structures of compound eyes, eight specimens (four males and four females) were used. The specimens were cleaned using an ultrasonicator for 3 min before decapitation of the head. The heads were immediately fixed in Bouin’s solution for 24 h and then dehydrated in graded series of ethanol (75%, 80%, 85%, 90%, 95%, and twice in 100% for 30 min in each concentration). The head samples were dried using a critical point dryer (Leica EM CPD300, IOZCAS, Beijing, China) for 30 min and mounted on a rotatable specimen holder. The samples were sputter-coated with gold layer for 33 nm (Leica EM SCD050, IOZCAS, Beijing, China) and examined in a scanning electron microscope (ESEM FEI Quanta 450, IOZCAS, Beijing, China), and micrographs were captured at an accelerating speed of 5–15 kV.

### 2.3. Transmission Electron Microscopy

To investigate the ultrastructural features of dorsal and ventral eyes of *D. mellyi*, eight samples (four males and four females) were used. The head was dissected to remove the compound eyes, which were immediately fixed in 0.1 M cacodylate buffered 5% glutaraldehyde (pH 7.4) for 24 h. The samples were then post-fixed in 1% osmium tetroxide for 24 h, then buffered in 0.1 M cacodylate buffer (pH 7.4) for 2 h. The samples were dehydrated in a graded series of ethanol (75%, 80%, 85%, 90%, 95%, and two times in 100% for 30 min in each concentration) and then dipped twice in pure acetone. The samples were infiltrated in a mixture of Acetone and Epon (3:1, 1:1, and 1:3 and pure Epon) at 60 °C for four days. The embedded samples were then trimmed and cut into ultra-thin sections using a diamond knife on an ultramicrotome.

The ultrathin sections obtained were stained with 2% aqueous uranyl lead acetate for 15 min and examined with a transmission electron microscope (Tencai spirit TEM, IBPCAS, Beijing, China).

### 2.4. Microcomputed Tomography and 3D Reconstruction

Two samples (one male and one female) of *D. mellyi* were used for tomography. The samples were decapitated, and the heads were dehydrated in a series of graded ethanol 75%, 80%, 85%, 90%, 95%, and three times in 100% (30 min in each concentration). The samples were dried in a critical point dryer (Leica EM CPD300), then mounted on an Eppendorf tube and scanned using the Xradia scanner (Zeiss MicroXCT-400, IOZCAS, Beijing, China) at a magnification of 4× and image capture at an interval of 10 s for 5 h.

The dataset is stored in the Institute of Zoology, Beijing, China, and is available for access through the corresponding author.

Amira software version 6.0.1 (Thermo Fisher Scientific, Waltham, MA, USA) was used in the segmentation of different structures of the compound eyes from the image stacks obtained through scanning. The segmented materials were imported to VG Studio Max 3.1 (Volume Graphics, Heidelberg, Germany) for rendering and visualization.

The volume rendering of different structures that make up the compound eyes were performed through Amira software version 6.0.1, and the final images were assembled through Adobe Photoshop version 21.2.1 (Adobe Inc., San Jose, CA, USA).

### 2.5. Determination of Wetting Properties

Six freshly captured *D. mellyi* samples were anesthetized, and heads were severed from the body to determine wetting properties of the eyes. To measure the contact angle, a water droplet (0.15 µL) was planted on the compound eyes with an Eppendorf micropipette. The images were captured using a digital camera mounted upon a horizontally oriented optical microscope. The images of contact angles were analyzed using ImageJ 1.53 software (National Institutes of Health, Bethesda, MD, USA). The contact angles were measured on both the right and left sides of the droplet. The data of dorsal and ventral eyes were analyzed to determine the standard error and plotted.

### 2.6. Data Analysis

The results from scanning electron microscopy were used to determine various parameters, including the number of facets, facetal area, diameter, and types of ommatidia. The cross-sectional and transverse micrographs at different depths of the compound eyes were used in calculating the area occupied by different internal structures of the compound eyes. The longitudinal sections were used to calculate the length of the different features of ommatidia and determine the number of retinular cells, primary pigment cells, secondary pigment cells, and mitochondria. Examination of the different parameters in scanning electron microscopy and transmission electron microscopy was performed with ImageJ 1.53 software.

Microcomputed tomography data were used in visualizing the structural organization of the dorsal and ventral eyes and determining the relative positioning of different features of the ommatidia.

## 3. Results

### 3.1. General Overview

**Figure 1:**
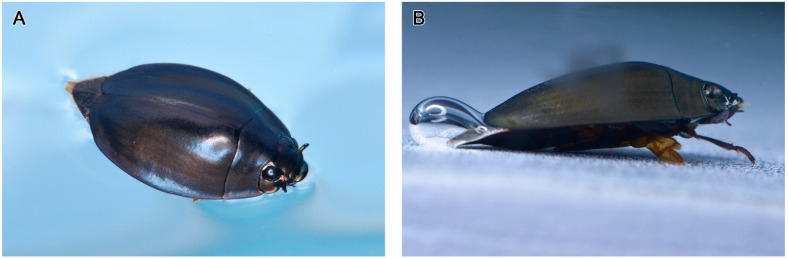
The ecological positioning of adult *D. mellyi*. (**A**) Individual floating on the surface of water. (**B**) Individual resting under the water surface.

**Figure 2:**
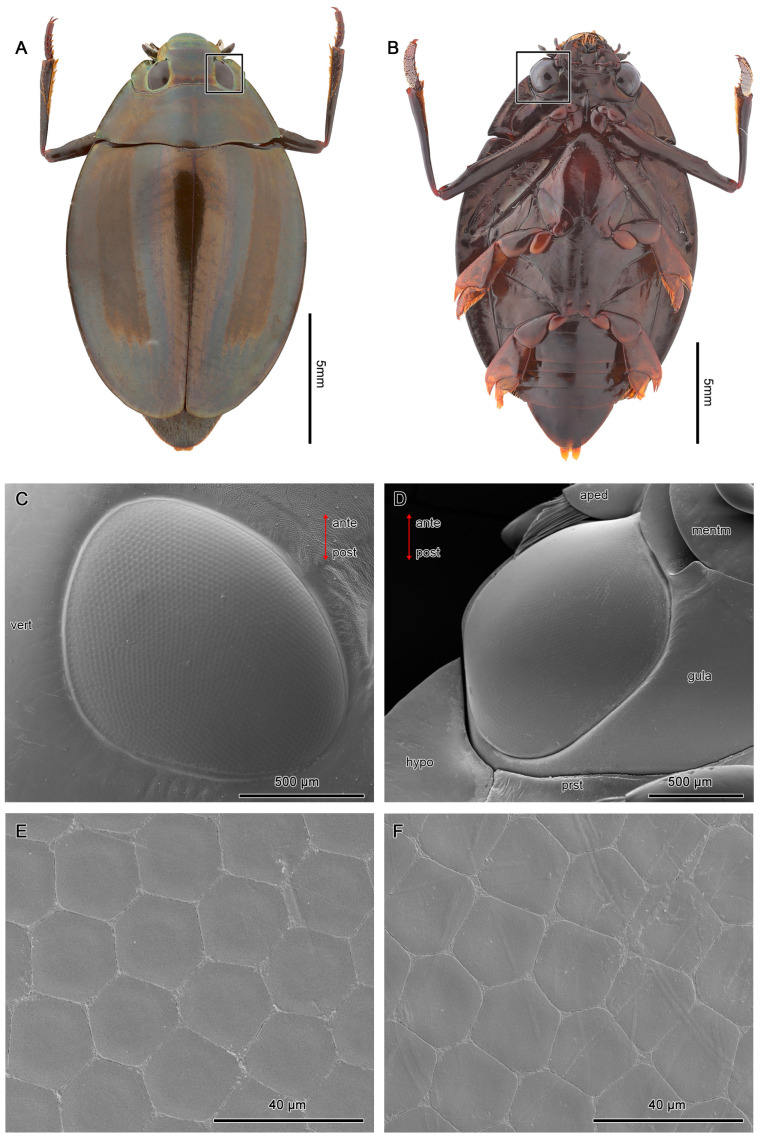
Exterior morphologies of the dorsal (**A**) and ventral eyes (**B**) of *D. mellyi* (squared). (**C**,**D**) SEM micrographs of the dorsal and ventral eyes, respectively. (**E**) SEM micrographs of facets at the center region, showing the regular hexagonal facets. (**F**) SEM micrographs of facets at the peripheral region, showing the pentagonal facets. Vert, vertex; hypo, hypopleuron; prst, prosternum; gula; mentm, mentum; aped, apedicel; ante, anterior; post, posterior.

Adult *D. mellyi* possess a pair of split compound eyes, which are composed of a pair of dark elliptical dorsal parts and a pair of dark and shiny grooved ovate ventral parts (Figure 1 and Figure 2).

Each of the dorsal eyes is made up of an average of 1913 ± 44.5 facets, while the ventral compound eyes contain an average of 3099 ± 86.2 facets (Table 1). The shape of facets varies from hexagonal at the center region to pentagonal at the peripheral regions of the compound eyes (Figure 2C,D). The 2 pairs of compound eyes are separated by an interocular bridge.

### 3.2. Three-Dimensional Reconstruction

Three distinct parts which form the ommatidial structure are observed in the reconstructed data of the dorsal and ventral eyes.

**Figure 3:**
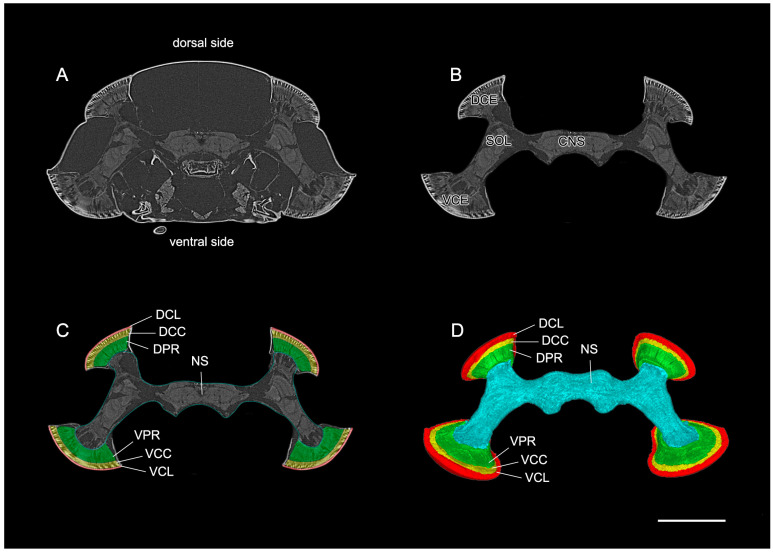
The direct rendering of the standard raw data of the split compound eyes of *D. mellyi*. (**A**) Virtual frontal 2D slice from the micro-CT data showing the position of the compound eyes in the head capsule. (**B**) Same slice as in (**A**) but cropped to include all optical and neuronal tissues. (**C**) The cropped slice with all optical regions of interest manually labeled. (**D**) Reconstructed surface model of an individual compound eyes and nervous system. DCE, dorsal compound eye; VCE, ventral compound eye; SOL, split optic lobe; CNS, central nervous system; DCL, corneal lens of dorsal eyes; DCC, crystalline cone of dorsal eye; DPR, photoreceptive region of dorsal eye; VCL, corneal lens of ventral eye; VCC, crystalline cone of ventral eye; VPR, photoreceptive region of ventral eye; NS, nervous system. Scale bar = 1000 μm.

**Figure 4:**
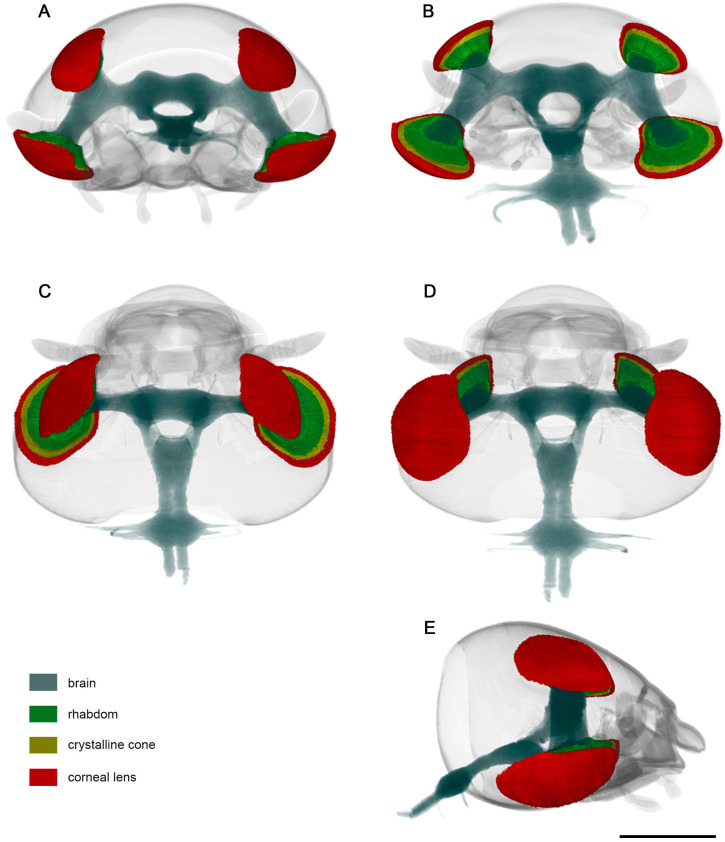
Standard-based shapes of surface reconstruction of visual system of *D. mellyi* from different orientations: (**A**) anterior; (**B**) posterior; (**C**) dorsal; (**D**) ventral; (**E**) lateral. Different colors code specific reconstructed compound eye structures. Scale bar = 1000 μm.

The corneal lens forms the outermost layer and shows a narrow, elongated, curved design that spreads from edge to edge, covering the crystalline cone (Figure 3 and Figure 4). The corneal lens becomes thinner towards the peripheral regions of the compound eyes.

Between the corneal lens and photoreceptive elements lies a crystalline cone whose thick structure conforms to the shape of the corneal lens. It spreads from edge to edge, covering the photoreceptive elements. Notably, the peripheral regions of the crystalline cone tend to curve inwards, taking the spherical shape of the compound eyes (Figure 3 and Figure 4).

The photoreceptive regions form a thick, curved structure whose shape conforms to the proximal design of the crystalline cone (Figure 3 and Figure 4).

### 3.3. Measurement of the Wetting Properties

Six beetle samples were used to test the wettability properties of the dorsal and ventral compound eyes. A drop of water (15 µL) was placed on the surface of the compound eyes, and the measurement of the angle was performed manually using ImageJ 1.53 software. The contact angle results of both regions of the compound eyes were not significantly different from each other. The mean average of dorsal and ventral eyes was 109.2° and 110.5°, respectively. Therefore, both dorsal and ventral compound eyes show a weak hydrophobic property (Figure S1). This finding aligns with the results reported by Blagodatski et al. (2014) [1].

### 3.4. Internal Organization

Each ommatidium in dorsal and ventral eyes in *D. mellyi* is made up of two distinct structures: the dioptric apparatus, comprised of the corneal lens and crystalline cone, and the photoreceptive elements, made up of eight retinular cells and their rhabdomeres. The corneal lens is in direct contact with the crystalline cone.

**Figure 5:**
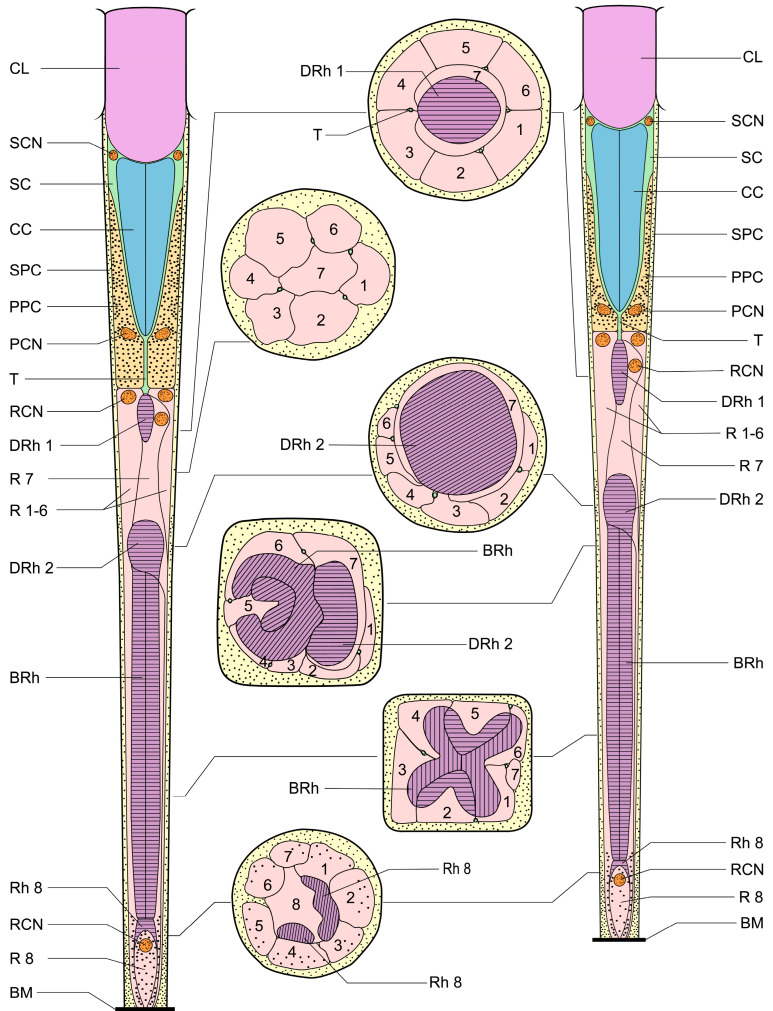
Schematic illustration of the dorsal and ventral eyes of *D. mellyi*. Ommatidium of ventral eyes (**left**) and dorsal eyes (**right**) with cross-sectional illustrations. CL, cornea lens; SC, Semper’s cells; SCN, nucleus of Semper’s cell; CC, crystalline cone; SPC, secondary pigment cells; PPC, primary pigment cells; PCN, nucleus of primary pigment cell; T, crystalline tracts; DRh 1, upper distal rhabdom; DRh 2, lower distal rhabdom; R, retinular cells; RCN, nucleus of retinular cell; BRh, basal rhabdom; Rh, rhabdomere; BM; basal membrane. The numbers in the cross-sectional illustration indicate the numbering of retinular cells.

Electron micrographs of both cross-sectional and longitudinal sections of dorsal and ventral eyes show a laminated corneal lens, as well as bullet-shaped crystalline cones equally contributed by four Semper’s cells. The cell bodies of Semper’s cells form a thin sheath enveloping the crystalline cones. The Semper’s cells and the crystalline cone are surrounded by the primary pigment cells and secondary pigment cells. The quadripartite Semper’s cells form direct contact with the retinular cells by a narrow extension called the crystalline tract, a characteristic that is essential for the transmission of light signals between the dioptric apparatus and the photoreceptive region. The retinular cells run down from the proximal regions of the crystalline cone to the basement membrane. A clear zone exists between the upper and lower distal rhabdom. Notably, significant differences in the morphology of the rhabdom and the arrangement of retinular cells are noted along the vertical axis (Figure 5).

#### 3.4.1. Dioptric Apparatus

**Figure 6:**
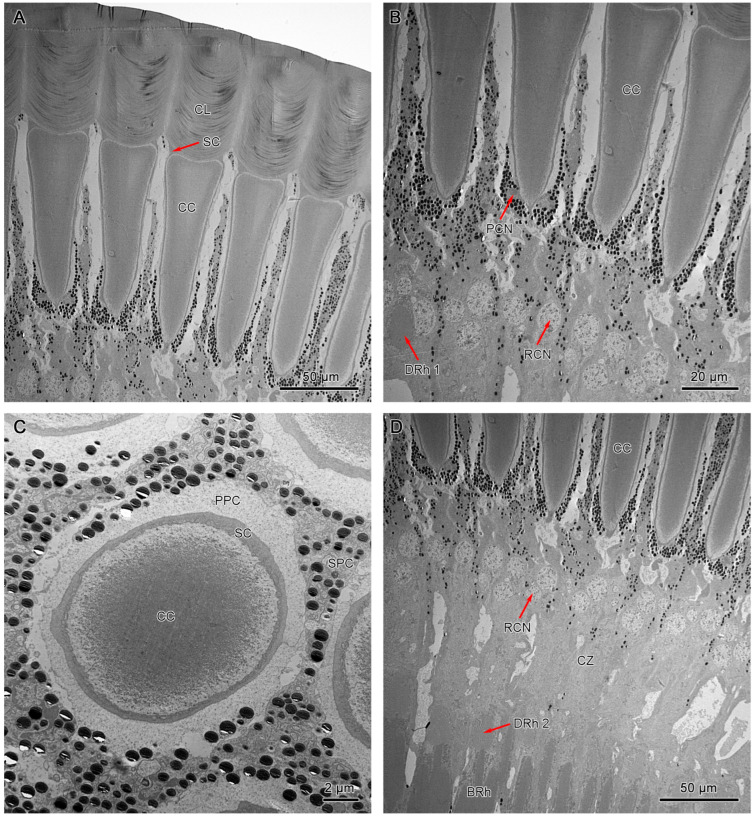
TEM micrograph of the dioptric apparatus of the ventral compound eyes. (**A**) Longitudinal section of the corneal lens and crystalline cone. (**B**) Longitudinal section shows crystalline cone with tract extensions. (**C**) Cross-section of crystalline cone shows the quadripartite eucone-type crystalline cone. (**D**) Longitudinal section of proximal region of ommatidia shows the crystalline cone, distal rhabdom, and clear zone. CL, cornea lens; SC, Semper’s cells; CC, crystalline cone; SPC, secondary pigment cells; PPC, primary pigment cells; PCN, nucleus of primary pigment cell; DRh 1, upper distal rhabdom; DRh 2, lower distal rhabdom; CZ, clear zone; BRh, basal rhabdom; RCN, nucleus of retinular cell.

**Figure 7:**
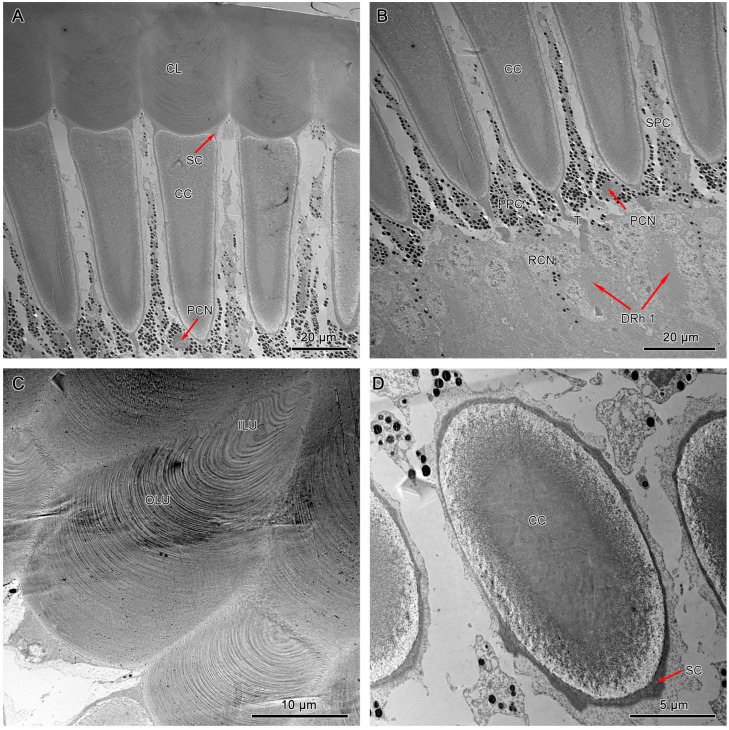
TEM micrograph of the dioptric apparatus of the dorsal compound eyes. (**A**) Longitudinal section of the corneal lens and crystalline cone. (**B**) Longitudinal section of the crystalline cone shows the thread extensions (T). (**C**) Transverse section of the corneal lens shows the layers with different electron densities. (**D**) Transverse section of the crystalline cone shows the quadripartite eucone-type crystalline cone. CL, cornea lens; CC, crystalline cone; SC, Semper’s cell; SPC, secondary pigment cells; PPC, primary pigment cells; PCN, nucleus of primary pigment cell; DRh 1, upper distal rhabdom; ILU, inner lens unit; OLU, outer lens unit.

Both dorsal and ventral eyes show a morphological uniformity in dioptric components. The laminated cornea shows a subconvex shape from the outside and a convex shape from the inside that tends to conform to the distal shape of the crystalline cone (Figure 6A and Figure 7A).

It measures 40–50 µm and 56–60 µm in length in dorsal and ventral eyes, respectively. Transverse sections of the corneal lens from both compound eyes exhibit some concentrations of lens units that vary in electron intensity (Figure 7C). These lens units are grouped into dense inner lens units and less dense outer lens units. Longitudinal sections of dorsal and ventral eyes show direct contact between the proximal region of the corneal lens and the bullet-shaped crystalline cone with the Semper’s cells positioned at the transition area (Figure 6A and Figure 7A).

The crystalline cones of both compound eyes are eucone-type and comprised of four wedge-shaped Semper’s cells (Figure 6C and Figure 7D). The four Semper’s cells run along the cone and reduce significantly in size, forming crystalline tracts (Figure 6B and Figure 7B). The average measurement of the crystalline cone in dorsal eyes is 50–62 µm in length and 10–20 µm in thickness. In ventral eyes, the average measurement of the crystalline cone is 70–75 µm in length and 12–18 µm in diameter.

#### 3.4.2. Pigment Cells

**Figure 8:**
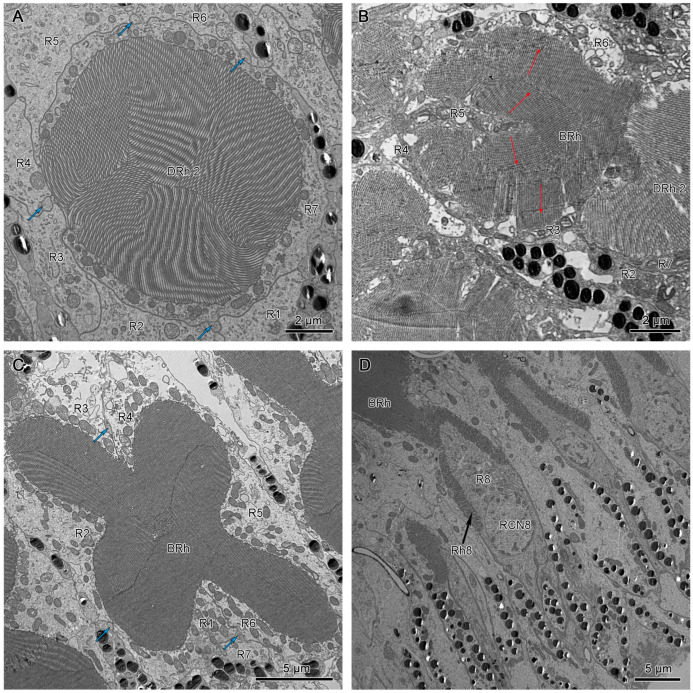
(**A**) Transverse section of the lower distal rhabdom of ventral eyes shows a round-shaped rhabdom in the distal region. (**B**) Transverse section of the transition region between the lower distal rhabdom and the basal rhabdom in dorsal eyes, the red arrows indicate the orientation of microvilli. (**C**) Transverse section of basal rhabdom of dorsal eye shows a cross-shaped rhabdom formed by retinular cells 1–6 (R1–R6) and an axon of retinular cell 7 (R7). (**D**) Longitudinal section of the proximal region of the basal rhabdom shows the rhabdom of retinular cell 8 (R8) that extends to the basement membrane. The blue arrows in (**A**,**C**) indicate the crystalline tracts between the retinular cells. DRh 2, lower distal rhabdom; BRh, basal rhabdom; RCN, nucleus of retinular cell; R, retinular cells; Rh, rhabdomere of corresponding retinular cell.

Longitudinal sections of dorsal and ventral eyes show a concentration of screening pigments that run between the crystalline cones of different ommatidia. Two primary pigment cells (PPC) surround the crystalline cone together, with pigment granules concentrating at the distal part. The secondary pigment cells (SPC) extend from the level of the crystalline cone all the way to the basement membrane. The pigment granules of SPC are barely present in the clear zone area. The number of SPC is hard to determine, probably 14 in each ommatidium (Figure 6B, Figure 7B, and Figure 8D).

#### 3.4.3. Photoreceptive Elements

**Figure 9:**
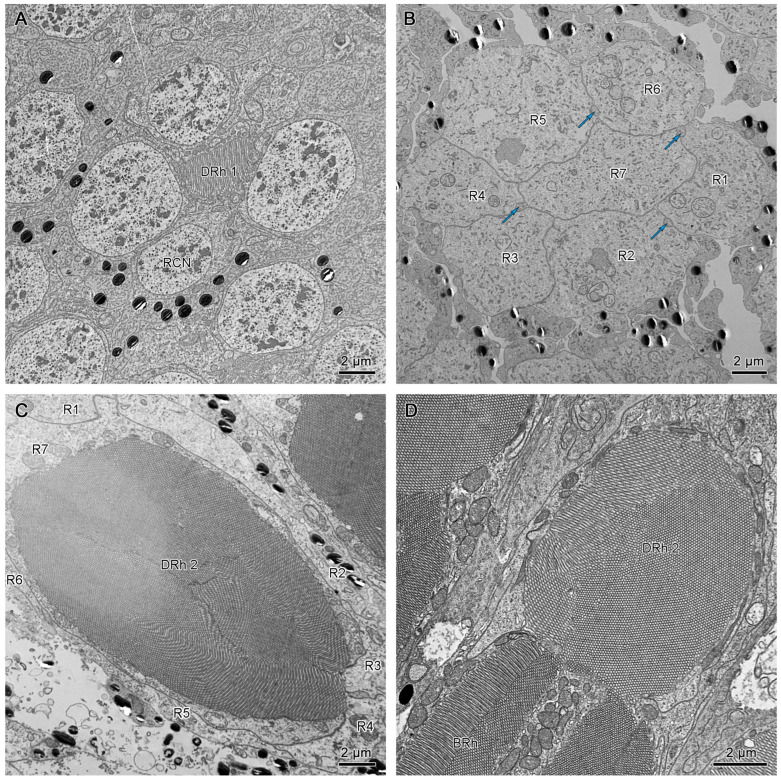
(**A**) Cross-section of the upper region of the distal rhabdom of retinular cell 7 (R7) of dorsal eye shows the upper distal rhabdom and nuclei of R1, R3, R4, and R6. (**B**) Cross-section of the clear zone region just beneath the upper distal rhabdom shows the cell bodies of retinular cells. The blue arrows indicate the crystalline tracts. (**C**) The proximal regions of distal rhabdom of ventral eyes. (**D**) Longitudinal section of the transition point between distal rhabdom and basal rhabdom in ventral eyes. DRh 1, upper distal rhabdom; DRh 2, lower distal rhabdom; R, retinular cells; RCN, nucleus of retinular cell; BRh, basal rhabdom.

The crystalline tracts extend downwards, forming a direct link with the circular to spindle-shaped upper distal rhabdom (DRh 1) that is contributed entirely by retinular cell 7 (R7). The upper distal rhabdomere is surrounded by the cell bodies of retinular cells 1–6 (R1–R6) (Figure 9A). On reaching the rhabdom region, crystalline tracts separate into four thin threads that run along the ommatidium to the basement membrane, passing through the intercellular space of R1/R2, R3/R4, R5/R6, and R6/R7, respectively (Figure 8A,C and Figure 9B). Each of the upper distal rhabdomeres receives light signals from a specific visual field. 

The upper distal rhabdom undergoes a substantial decrease in size towards the distal end, finally disappearing in the clear zone region. The clear zone area is comprised of the cell bodies of the retinular cell 1–6 (R1–R6) and surrounding secondary pigment cells. Four crystalline cone tracts are also observed in the region (Figure 9B).

The distal rhabdom reappears just below the clear zone, forming the lower distal rhabdom (DRh 2), which displays greater width and a varied morphological shape towards the basal rhabdom (Figure 8A and Figure 9C). The round to spindle-shaped lower distal rhabdom covers a larger surface area than the upper distal rhabdom, but this gradually decreases towards the distal region of the basal rhabdom. The lower distal rhabdom is in direct contact with the basal rhabdom (BRh) (Figure 9D).

**Figure 10:**
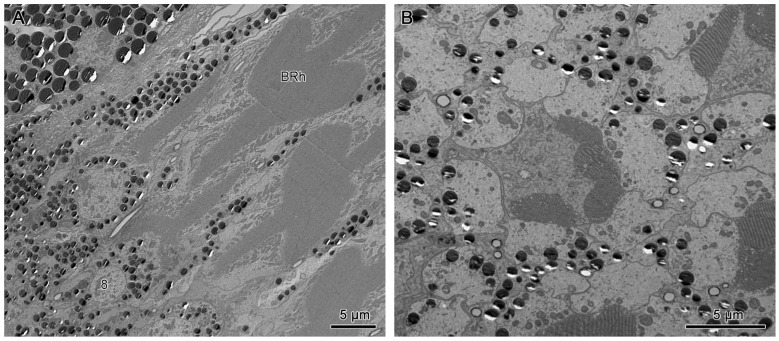
(**A**) Longitudinal section of the proximal region of the basal rhabdom. (**B**) Transverse section in the basal part of ommatidium, shows the rhabdom of retinular cell 8 (R8). BRh, basal rhabdom; Rh 8, rhabdomere 8; R, retinular cell; RCN, nucleus of retinular cell; BM, basement membrane; AR, axons of retinular cells.

Unlike the distal rhabdom, the basal rhabdom is entirely composed of the rhabdomeres of retinular cells 1–6 (R1–R6), while the retinular cell 7 (R7) appears as an axon between retinular cells 1 and 6 (R1 & R6) (Figure 8C). Despite the variation in length and width, both dorsal and ventral eyes show morphologically similar basal rhabdom. The rhabdomeres of retinular cells 1–6 (R1–R6) jointly form the cross-shaped rhabdom that pair as 2/5, 2/4, and 1/6 according to the photoreceptor subtype classification of Friedrich et al. [22], which run down to connect with the rhabdomere of retinular cell 8 (R8) (Figure 8D and Figure 10A). 

**Figure 11:**
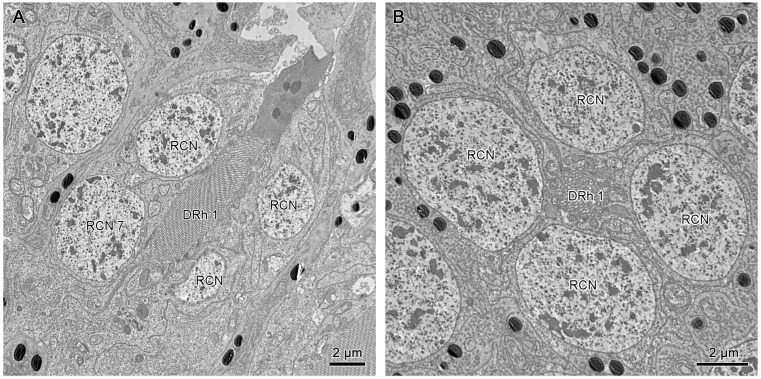
(**A**) Longitudinal section of upper distal rhabdom, showing the nuclei of R7 and other retinular cells. (**B**) Transverse section of upper distal rhabdom and four nuclei of other retinular cells at the same level; DRh 1, upper distal rhabdom; RCN, nucleus of retinular cell.

The longitudinal sections show that the nuclei of R1–R7 are located by the upper distal rhabdom at the distal region of the clear zone area, roughly organized into two rows (Figure 6B,D). More nuclei are present in the upper row, while the nucleus of R7 lies in the lower row (Figure 11A). According to Friedrich et al. [22], the nucleus position of R1/R6 is coordinated with R3/R4, while R2/R5 has a different nucleus position. Thus, we determine that the nuclei of R1, R6, R3, and R4 are located in the upper row (Figure 9A and Figure 11B). By contrast, the nuclei of R2 and R5 lie in the lower row, like R7.

The small retinular cell 8 (R8) is easily identifiable by its cell nucleus and is located just above the basement membrane, which contributes to a minute rhabdom that is in contact with the proximal region of the basal rhabdom (Figure 8D and Figure 10B). The axons of all the retinular cells run in compact bundles through the convex basement membrane into the optic lobes of the brain (Figure 10A).

Individual rhabdomeres are made up of closely packed microvilli. The morphology of these structures present in the dorsal and ventral eyes is observed to differ with respect to their location within the ommatidium. The upper distal rhabdom shows unidirectional microvilli (Figure 9A). However, a slight degree of variation in the angle of orientation is observed over several ommatidia. 

The microvilli of the lower distal rhabdom show confused orientation (Figure 8B and Figure 9C).

**Figure 12:**
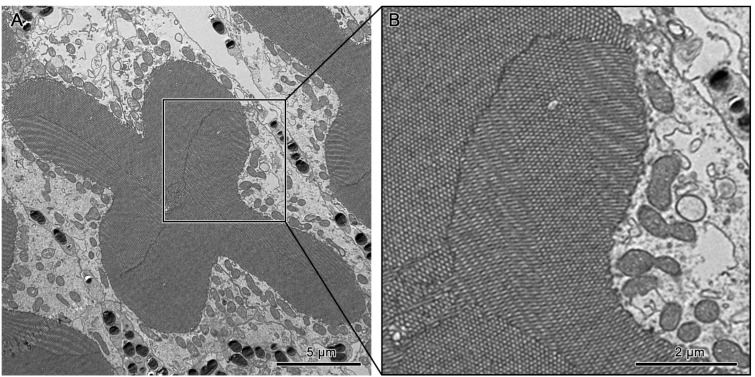
(**A**) Transverse section of the basal rhabdom. (**B**) A section of the transverse section of the basal rhabdom shows the banded arrangement of microvilli in retinular cells 3 and 6 (R3 & R6).

The cross-shaped rhabdom of the basal rhabdom shows multi-directional microvilli. The microvilli of retinular cells 3/4 and 1/6 (R3/R4 & R1/R6) pairs show a parallel orientation, whereas the 2/5 pair is made up of bands of microvillus that show a banded arrangement of the microvilli (Figure 8C and Figure 12A,B). The microvilli of rhabdom of retinular cell 8 (R8) orientate confusedly (Figure 8D and Figure 10B).

Notably, both the dorsal and ventral compound eyes show a similar arrangement of microvilli, including in the upper distal rhabdom, the lower distal rhabdom, the basal rhabdom, and the rhabdomere of the eight retinular cells.

## 4. Discussion

### 4.1. General Overview

Over time, the anatomical studies of compound eyes in insects began to revolve around the use of extensive histological procedures: light microscopy, scanning electron microscopy, and transmission electron microscopy. These procedures are very effective and provide high-resolution images but are destructive and labor-intensive [23]. The development and application of micro-CT to investigate the micro-internal structures of insects is a major milestone towards a detailed study of morphological structures and offers potential research into their development, evolution, and functions [24,25]. Anatomy experts used micro-CT in the study of head morphology [26,27], thoracic anatomy [28], the brain [25], reproductive parts [29], muscles [30], and the general anatomy of miniature structures [31,32]. Our study explored the reconstruction, visualization, and virtual analysis of the morphology of split compound eyes in whirligig beetles, supplementing advanced microscopy techniques.

### 4.2. Dioptric Apparatus

Different groups of insects show morphological and functional modifications in their compound eyes, often related to different environmental factors such as light intensity. In addition, previous studies link functional modifications of compound eyes to differing photosensitivity, color discrimination, spatial resolution, maximum acuity, and polarized vision [33,34,35].

The corneal lens in both dorsal and ventral eyes is laminated with a thick layer of chitin that shows well-documented cuticular regions of different electron intensities. Past studies interpreted this as an adaptation crucial for protecting the compound eyes from mechanical injuries. In addition, corneal laminations are described as light enhancers and regulators of light passing to the photoreceptive regions [19,36,37,38]. The dorsal and ventral eyes exhibit similar wettability, consistent with the findings reported by Blagodatski et al. (2014) [1].

In the proximal region of the corneal lens lies four Semper’s cells that form the crystalline cone. The proximity between these structures ensures improved transmission of light signals from the dioptric apparatus to photoreceptive regions [39,40]. The crystalline cone of *D. mellyi* is of the eucone type, as in other adephagan species [41]. The crystalline cones in both dorsal and ventral eyes are bullet-shaped with wide distal regions that decrease significantly, forming crystalline tracts. This modification enhances the accommodation of light directed from the corneal lens while the crystalline tracts function as light guides toward the photoreceptive regions [42,43]. 

### 4.3. Photoreceptive Elements

The close anatomical similarity between the photoreceptive elements of the dorsal and ventral eyes indicates a similar optical mechanism.

The photoreceptive elements in whirligig beetles show a tri-tiered rhabdom made up of a distal, basal, and eight retinular cell rhabdom. The direct link between the crystalline tracts and upper distal rhabdom is likely to serve as an optical modification, specifically as a light guide [38]. 

Below the upper distal rhabdomere is a clear zone that is composed of the cell bodies of retinular cells 1–6 (R1–R6), crystalline tracts, and secondary pigment cells. The clear zone serves as the diagnostic feature, indicating both dorsal and ventral eyes are superposition eyes. This area indicates the proximal region of the ommatidium receives light from different facets, thereby collecting more light in a poorly lit environment [44]. In addition, a clear zone also improves photosensitivity by several orders of magnitude at the expense of lowering the acuity of the eyes [45,46]. The circular to spindle-shaped lower distal rhabdom in the dorsal and ventral eyes has a larger cross-sectional area than the upper distal rhabdom. The increased cross-sectional area of this region is linked to increased photon capture from an individual ommatidium. At the transition region between the distal and basal rhabdom is an enlarged rhabdom made up of round-shaped rhabdom and emerging basal rhabdom. The transition zone between the lower distal rhabdom and basal rhabdom shows an increased surface area and possibly serves as an adaptation to improved photosensitivity. In addition, the multidirectional orientation of microvilli in this rhabdom indicates the polarization sensitivity between the two sets of microvilli probably reduces or cancels out, thereby reducing polarization sensitivity. This phenomenon is reported to be crucial in the enhancement of color discrimination [47,48,49].

The basal rhabdom is composed of retinular cells 1–6 (R1–R6) and occupies the largest proportion of the compound eyes in both dorsal and ventral eyes. Their long length is crucial in increasing photon capture. Transverse sections of this region show a fused cross-shaped rhabdom. The rhabdomeres of different retinular cells occur in pairs; R1/R6 and R3/R4 pairs show a similar orientation of the microvilli, while the R2/R5 pair shows a bidirectional orientation to the latter. The close fusion to the rhabdomere in this region indicates a close interaction of rhabdomere components, leading to electrical and optical coupling. This phenomenon has been described in other insects as crucial for fine-grain color vision due to a reduced field of view [12,49,50].

The photoreceptor structure of *D. mellyi* is composed of a distal rhabdom provided by R7, a basal rhabdom provided by R1–R6, and Rh8. This structural arrangement bears similarities to *Dytiscus marginalis* [51] and certain cicindelid beetles [52], suggesting a common structure within Adephaga. However, we also discovered a distinct feature in Adephaga. For instance, the compound eyes of *Agabus japonicus*, a dytiscid beetle species, exhibit an apposition type with highly directional and orthometric microvilli in the rhabdom [53]. In contrast, the microvilli in *D. mellyi* appear noticeably disoriented, indicating a lack of polarized sensitivity. Furthermore, we observed an intergeneric difference in the rhabdom structure. In *Gyrinus substriatus*, the upper and lower parts of the distal rhabdom are connected by attenuated rhabdomeres consisting of a small number of microvilli [54]. In comparison, no microvilli were observed between the upper and lower parts of the distal rhabdom in *D. mellyi* (Figure 9B).

## 5. Conclusions

This study described the functional morphology of split compound eyes in the whirligig beetle *D. mellyi*. Scanning electron microscopy, transmission electron microscopy, and microcomputed tomography reveal several morphologically relevant ommatidial features of both dorsal and ventral eyes. The split compound eyes exhibit optical structures suggestive of an adaptation to low-light environments. The multidirectional orientation of microvilli in the fused rhabdom of the dorsal and ventral eyes suggests that the basal rhabdom of whirligig beetles increases photon capture and also allows color discrimination. Despite differences in the environment surrounding the dorsal and ventral compound eyes, they exhibit similar functional modifications. This description and analysis of the morphological aspects of the ommatidia of dorsal and ventral eyes provide a defined background to more research into the evolution of different ultrastructures in split compound eyes of whirligig beetles.

## Figures and Tables

**Table 1:** Measured external parameters of the dorsal and ventral compound eyes of *D. mellyi*.

Parameters	N	Units	Average
Body size	8	mm	14.5 ± 2.0
Facet number in dorsal eyes	8	-	1913 ± 44.5
Facet number in ventral eyes	8	-	3099 ± 86.2
Compound area in dorsal eyes	8	mm^2^	1.22 ± 0.08
Compound area in ventral eyes	8	mm^2^	1.66 ± 0.12
Hexagonal facet area	40	µm^2^	408 ± 12.7
Pentagonal facet area	40	µm^2^	436.17 ± 43.9

Note: (N) The number of samples (average) is given as the mean and standard deviation of each measurement.

## Data Availability

All analyzed data are available in this paper. However, the raw micro-CT data can be made available upon request to the corresponding author.

## Supplementary Materials

The following supporting information can be downloaded at: https://www.mdpi.com/article/10.3390/insects15020122/s1, Figure S1: (**A**) The box plot shows a comparison of contact angles between the dorsal and ventral eyes of *D. mellyi*. The difference between the contact angles between regions of the compound eyes was insignificant. (**B**) Representative photos of water droplets on the surface of dorsal eyes and ventral eyes respectively.
