# Supplementary material for: Functional Anatomy of Split Compound Eyes of the Whirligig Beetles Dineutus mellyi (Coleoptera: Gyrinidae)
Source: Insects. 2024 Feb 7;15(2):122. doi: 10.3390/insects15020122 (PMC10889679; doi:10.3390/insects15020122)
Supplement: Supplementary file 1 [file insects-15-00122-s001.zip › insects-2798579-supplementary.pdf]

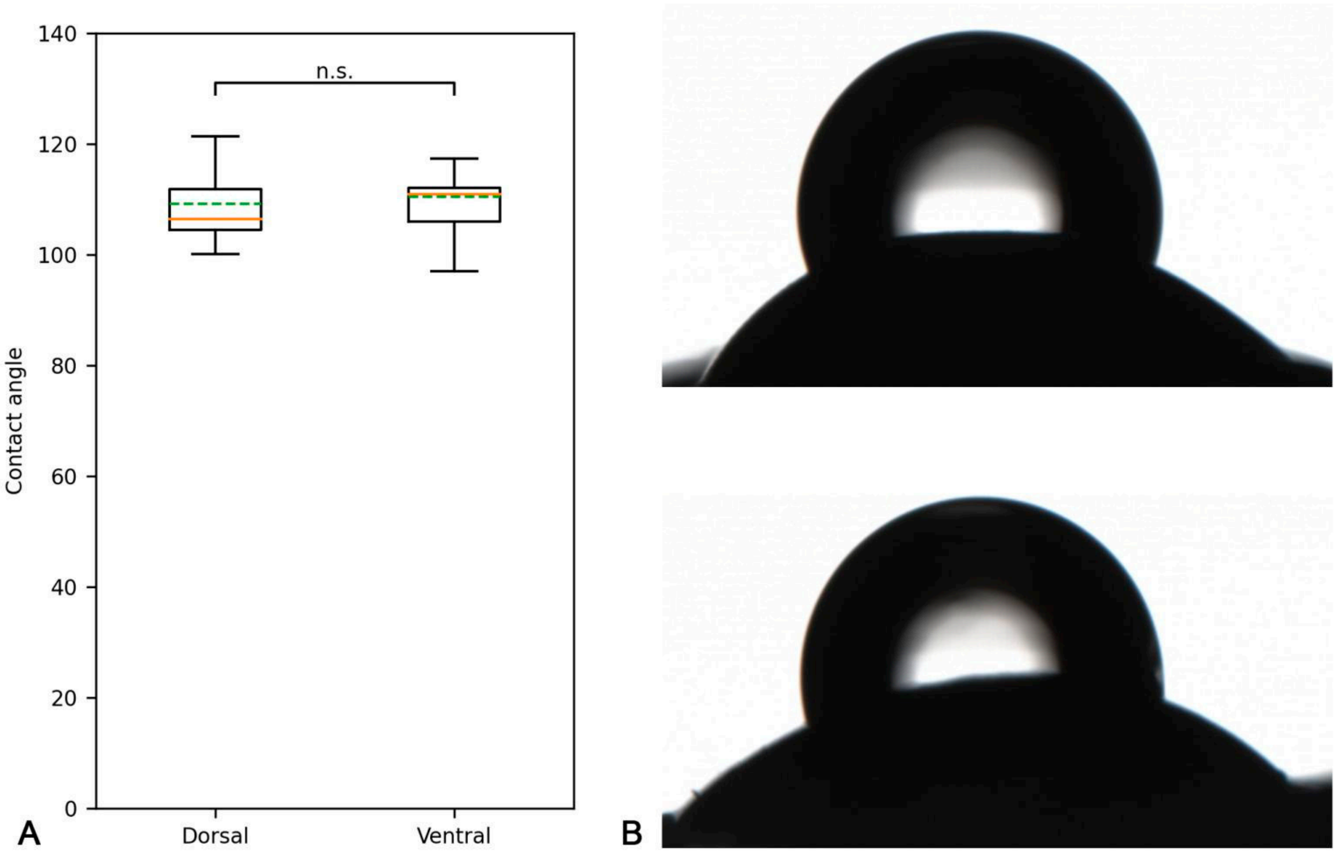

Figure S1. (A) The box plot shows a comparison of contact angles between the dorsal and ventral eyes of *D. mellyi*. The difference between the contact angles between regions of the compound eyes was insignificant. (B) Representative photos of water droplets on the surface of dorsal eyes and ventral eyes respectively.
